# Amputation of the Thigh under Hyoseine-Morphine-Cactin Anesthesia

**Published:** 1907-05

**Authors:** Henry C. Ebert

**Affiliations:** Assistant Surgeon in the Public Health and Marine Hospital Service


### Abstracts and Selections

### Amputation of the Thigh under Hyoscine-Morphine-Cactin Anesthesia.

BY HENRY C. EBERT, M. D.,
Assistant Surgeon in the Public Health and Marine Hospital Service.

I wish to report the successful use of the hyoscine, morphine and cactin combination as a general anesthetic in an amputation of the thigh in the upper one third.

The tablets used were those put up by the Abbott Alkaloidal Company, and contained Hyoscine Hydrobromide, gr. 1-100, Morphine Hydrobromide, gr. ¼, Cactin, gr. 1-67. Injections (hypodermic) were given two hours, one hour and a half before operation. Anesthesia was ideal and complete throughout operation and for several hours afterward. No ill effects whatever were noticed at any time. Muscular relaxation was not so complete as in ether or chloroform anesthesia so that after the operation no subsequent contraction of flaps took place, and there was no more tension on the stitches afterward than at the time they were put in.

If this anesthetic will work in all cases as well as it did in this and numerous others reported in the medical journals, it would appear to be the ideal anesthetic for field use and emergency work where one may be short handed, as it does away entirely with the anesthetist and the space and care necessary in the transportation of ether or chloroform.

The absence of inconvenient after-effects is a most valuable feature of this preparation in field work, while the ability to perform serious operations promptly is of particular advantage; but of equal utility in active service is the possibilty of securing complete rest and anesthesia in cases of injuries too extensive to permit of immediate operative attention, such as in visceral injuries of the abdomen, chest or head. It seems a good thing for the military surgeon and should come into favor with him.—*From the Military Surgeon, Journal of the Association of Military Surgeons of the United States.*

